# Stockpiling Ventilators for Influenza Pandemics

**DOI:** 10.3201/eid2306.161417

**Published:** 2017-06

**Authors:** Hsin-Chan Huang, Ozgur M. Araz, David P. Morton, Gregory P. Johnson, Paul Damien, Bruce Clements, Lauren Ancel Meyers

**Affiliations:** The University of Texas at Austin, Austin, Texas, USA (H.-C. Huang, G.P. Johnson, P. Damien, L.A. Meyers);; University of Nebraska, Lincoln, Nebraska, USA (O.M. Araz);; University of Nebraska Medical Center, Omaha, Nebraska, USA (O.M. Araz);; Northwestern University, Evanston, Illinois, USA (D.P. Morton);; Department of State Health Services, Austin (B. Clements);; Santa Fe Institute, Santa Fe, New Mexico, USA (L.A. Meyers)

**Keywords:** Ventilators, optimization, pandemic, influenza, viruses, Texas, United States

## Abstract

In preparing for influenza pandemics, public health agencies stockpile critical medical resources. Determining appropriate quantities and locations for such resources can be challenging, given the considerable uncertainty in the timing and severity of future pandemics. We introduce a method for optimizing stockpiles of mechanical ventilators, which are critical for treating hospitalized influenza patients in respiratory failure. As a case study, we consider the US state of Texas during mild, moderate, and severe pandemics. Optimal allocations prioritize local over central storage, even though the latter can be deployed adaptively, on the basis of real-time needs. This prioritization stems from high geographic correlations and the slightly lower treatment success assumed for centrally stockpiled ventilators. We developed our model and analysis in collaboration with academic researchers and a state public health agency and incorporated it into a Web-based decision-support tool for pandemic preparedness and response.

Diligent preparation and effective countermeasures are critical to mitigating future influenza pandemics. The 1918 influenza pandemic, the most severe in recent history, resulted in ≈50 million deaths globally, of which nearly 675,000 occurred in the United States (*1*). The 1957 and 2009 pandemics were less severe, causing ≈70,000 and 9,000–18,000 US deaths, respectively (*1*). The US Department of Health and Human Services (HHS) estimated (*2*) that 865,000 US residents would be hospitalized during a moderate pandemic (as in 1957 and 1968) and 9.9 million during a severe pandemic (as in 1918).

When severe influenza outbreaks cause high rates of hospitalization, a surge of medical resources is required, including critical care supplies, antiviral medications, and personal protection equipment. Given uncertainty in the timing and severity of the next pandemic, as well as the time required to manufacture medical countermeasures, stockpiling is central to influenza preparedness (*3*). However, difficulty in forecasting and limited public health budgets often constrain decisions about sizes, locations, and deployment of such stockpiles**.**

Mechanical ventilators are essential for treating influenza patients in severe acute respiratory failure. Substantial concern exists that intensive care units (ICUs) might have insufficient resources to treat all persons requiring ventilator support. Prior studies argue that current capacities are insufficient to handle even moderately severe pandemics and that sentinel reporting and model-based decision-making are critical for managing limited resources (*4*–*6*). For this reason, the United States has stockpiled mechanical ventilators in strategically located warehouses for use in public health emergencies, such as an influenza pandemic. The Centers for Disease Control and Prevention (CDC) manages this Strategic National Stockpile (SNS) and has plans for rapid deployment to states during critical events (*7*).

However, SNS ventilators might not suffice to meet demand during a severe public health emergency. In 2002, the SNS included ≈4,400 ventilators (*8*,*9*), and 4,500 SNS ventilators were added during 2009 and 2010. The American Association for Respiratory Care suggested the SNS inventory should increase to at least 11,000–16,000 ventilators in preparation for a severe influenza pandemic (*10*). The American Association for Respiratory Care and CDC (*11*) provide training on 3 types of SNS ventilators—LP10 (Covidien, Boulder, CO, USA); LTV1200 (CareFusion, Yorba Linda, CA, USA); and Uni-vent Eagle 754 (Impact Instrumentation, Inc., West Caldwell, NJ, USA)—to ensure proper use nationwide. In addition to the nationally held SNS, some US states maintain their own stockpiles.

Successful deployment of central ventilator stockpiles, whether federal or state, requires rapid distribution to healthcare facilities with patients in need, along with adequate bed space, requisite supplies, and trained personnel (*12*–*14*). Robust methods for sizing and locating ventilator stockpiles have not yet been developed (*15*). Wilgis (*16*) discussed the relative merits of central stockpiling of ventilators to be distributed during an emergency versus distributing ventilators to hospitals a priori. Centralized stockpiles benefit from better inventory tracking, more timely repairs, and superior allocation of a limited resource, but hospital-based supplies facilitate staff training, enable immediate use, and avoid the cost and logistical challenges of central storage and deployment.

We developed an optimization framework for allocating mechanical ventilators to central and local stockpiles to ensure adequate surge capacity during a future pandemic. This data-driven method considers the trade-off between risk and stockpiling cost, where risk is measured 2 ways: expected value of unmet demand (EUD; number of influenza patients not receiving required ventilation) and probability of unmet demand (PUD; probability at least 1 patient does not receive required ventilation). For a given set of healthcare providers in a region, we determined the optimal number of mechanical ventilators to stockpile centrally and at each provider site.

As a case study, we considered the US state of Texas under mild, moderate, and severe influenza pandemic scenarios. Based on the Texas Department of State Health Services (DSHS) response to the 2009 influenza A(H1N1) pandemic and planning efforts for future pandemics, we considered stockpiling across 9 sites: a centrally held state stockpile and local stockpiles in each of Texas’ 8 health service regions (HSRs; Technical Appendix Figure 1). We implemented this model in a Web-based decision-support tool for DSHS (*17*).

## Methods

**Figure 1:**
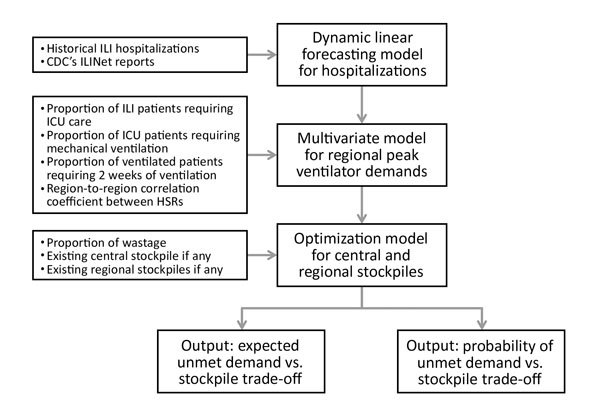
Overview of methods for projecting the need to stockpile ventilators for an influenza pandemic, Texas, USA. First, a forecasting model was used to estimate weekly hospitalizations at each site on the basis of historical ILI hospitalization data and CDC ILINet reports. Second, 3 additional factors, along with a spatial correlation coefficient, were used to form a probability distribution for peak-week ventilator demand at each site. Third, an optimization model was solved to determine local and central stockpile allocations and generate trade-off curves between the expected unmet demand and total stockpile and between the probability of unmet demand and total stockpile. CDC, Centers for Disease Control and Prevention; HSR, health service region; ICU, intensive care unit; ILI, influenza-like illness.

Our approach had 3 stages (Figure 1). First, we estimated the weekly influenza-related hospitalizations at each site using an adaptive time-dynamic forecasting model. Second, we estimated the number of patients requiring ventilation at each site during the peak week on the basis of published estimates of the proportion of hospitalized influenza patients requiring mechanical ventilation. Finally, we allocated ventilators at minimum cost to achieve a specified level of preparedness through a mathematical optimization model. That model assumed centrally stockpiled ventilators have slightly lower treatment rates than locally held ventilators. In the Texas case study, we estimated hospitalizations under a mild scenario by fitting the forecasting model to data from the 2009 influenza A(H1N1) pandemic, and then we scaled the estimates to simulate moderate and severe pandemics. We summarize our optimization model and forecasting methods and provide details in the online Technical Appendix.

### Optimization Model for Ventilator Stockpiling

Using a 2-stage model, we optimized the allocation of ventilators to a central stockpile and several local stockpiles (at healthcare facilities) to ensure that all sites had sufficient surge capacity to manage the peak of an influenza pandemic. We considered the trade-off between unmet ventilator demand (risk) and the cost of stockpiling ventilators (assuming cost is proportional to number of ventilators) and minimized cost while limiting risk to a specified threshold. We analyzed the risk–cost trade-off by solving a family of optimization models, across a range of risk thresholds.

We assumed the following: each stockpiled ventilator is both child- and adult-capable, will be used to treat at most 1 patient during peak demand, and will not be used for noninfluenza patients; stockpiles were established before the pandemic, and centrally held ventilators can be deployed only once to a site with excess demand (i.e., no redeployment is allowed, even though influenza peaks might be asynchronous across sites); patients requiring ventilatory support cannot move between sites; locally held ventilators are immediately and successfully administered to on-site patients requiring care, and centrally held ventilators incur wastage (i.e., a reduced fraction are successfully deployed to healthcare sites upon demand); patients at all sites have equal priority; and consumable ventilator supplies, requisite staffing, and space are in sufficient supply. The optimization model considers expected unmet demand, and we calculated the probability of unmet demand post hoc, as a secondary risk measure.

### Texas Case Study

We assumed that ventilators can be stockpiled centrally by the Texas DSHS or locally by hospitals in Texas’ 8 HSRs (Technical Appendix Figure 1). We further assumed that local stockpiles within an HSR are available throughout the HSR by movement of either ventilators or patients among healthcare facilities; that is, any patient within an HSR requiring ventilatory support has access to available ventilators within that HSR. To model peak ventilator demand across Texas’ 8 HSRs under different pandemic scenarios, we 1) estimated the region-to-region (HSR-to-HSR) correlation in peak-week ventilator demand on the basis of 2003–2008 seasonal influenza hospitalization data and 2009 pandemic hospitalization data; 2) generated probabilistic estimates of peak-week influenza-related hospitalizations by fitting our forecasting model to a baseline (mild) pandemic scenario estimated from 2009 pandemic data; 3) used the estimates derived in steps 1 and 2 to estimate the numbers of influenza patients requiring mechanical ventilation at the pandemic peak in each HSR; and 4) generated moderate and severe pandemic scenarios by scaling the peak demand estimates of the mild scenario.

### Texas Influenza Data

We obtained weekly Texas hospital discharge data for 2003–2009, filtered for International Classification of Diseases, Ninth Revision, codes 487 and 488, corresponding to influenza-like-illness (ILI), and aggregated by HSR. These data comprised all Texas hospitals except those in counties with populations <35,000, those with <100 hospital beds, and those that do not accept insurance or government reimbursement. The number of ILI-related hospital discharges during the 2009 pandemic (April–December 2009) totaled 29,459. We assessed the validity of this International Classification of Diseases, Ninth Revision–based filter for influenza through comparison with CDC (*18*) and Texas DSHS (*19*) reports. We summarize the parameters we used to estimate peak ventilator demand under different pandemic scenarios (Table 1) and outline the data and methods used to estimate these parameters.

**Table 1:** Parameters for estimating peak-week ventilator demand in mild, moderate, and severe influenza pandemics, Texas, USA*

Parameter	Mild (2009-like)	Moderate (1957- and 1968-like)	Severe (1918-like)	Source
Hospitalization scaling over mild	1	3.14	36	(*2*,*21*)
Proportion of hospitalized ILI patients requiring ICU care	0.2	0.25	0.25	(*2*,*19*,*21*,*22*,*23*)
Proportion of ICU patients requiring ventilation	0.5	0.5	0.5	(*2*,*21*,*22*)
Proportion of ventilated patients requiring 2 weeks of ventilation	0.4	0.4	0.4	(*22*)
Region-to-region correlation for peak-week demand	0.7	0.7	0.7	Estimated

*ICU, intensive care unit; ILI, influenza-like illness.

We also analyzed data from the CDC ILINet, which tracks weekly outpatient visits related to ILI. CDC guidelines define ILI as fever of at least 100°F and cough and/or sore throat in the absence of a known cause other than influenza. A network of 2,400 sites (health departments, laboratories, vital statistics offices, healthcare providers, and emergency departments) in the 50 states reports to ILINet, and we obtained weekly reports during the 2009 H1N1 pandemic for Texas, aggregated by HSR. Finally, Texas DSHS provided data on the 3,730 ventilators stockpiled in Texas in 2009 (Technical Appendix).

### Region-to-Region Correlation in Peak Hospitalizations

For each of the 6 influenza seasons in years starting 2003–2008 and the 2009 pandemic, we calculated peak-week ILI hospitalizations requiring ventilation in each HSR. Across all 28 pairs of HSRs, the average correlation in peak ventilator demand was 0.72 ± SD 0.23 (range 0.22–0.98). One HSR, with <3% of total hospitalizations during 2009, had pairwise correlations as low as 0.22, but all other pairs of HSRs had coefficients >0.50. We found similar spatiotemporal correlations in hospitalizations when we estimated pairwise HSR-to-HSR correlations for various values of the proportion of ventilated patients requiring 2 weeks (rather than 1 week) of ventilation, and weekly numbers of ILI hospitalizations requiring ventilation, throughout the 2003–2008 influenza seasons and the 2009 pandemic. Given this consistent statewide synchrony in epidemic intensity, we made the simplifying assumption that peak hospitalizations in all HSRs were correlated at a pairwise level of 0.70.

### Forecasting Model for Hospitalizations

We used a dynamic linear forecasting model (Technical Appendix), which provides a powerful method for capturing system uncertainty when numerous dynamic factors influence a system (*20*). Although hospitalizations could be forecast only on the basis of historical ILI data, our approach can incorporate additional predictors, such as the most recent ILINet reports, to better represent demand uncertainty. Our forecasting method estimated weekly influenza-related hospitalizations in the 8 HSRs for 2009 pandemic–like scenarios, using CDC ILINet influenza A(H1N1)pdm09 weekly reports as a predictor, from the week ending April 4, 2009, through the week ending December 26, 2009. To account for seasonality, we assumed 5 distinct time periods (September–October, November–December, January–February, March–April, and May–August). We also considered other candidate variables, such as school calendars, humidity, and Google Flu Trends, but these did not substantially improve peak estimates.

### Estimating Regional Ventilator Demand

To estimate regional ventilator peak-week demand, we integrated our weekly forecasts of influenza hospitalizations in each region, the spatial correlation in peak-week demand for ventilators, and 3 additional factors: 1) the proportion of hospitalized ILI patients requiring ICU care, 2) the proportion of ICU patients requiring ventilation, and 3) the proportion of ventilated patients requiring 2 weeks of ventilation (rather than 1). To model “spillover” demand of patients requiring 2 weeks of ventilation, we used week-to-week correlations in influenza hospitalization (Technical Appendix Table 2).

#### Proportion of Hospitalized ILI Patients Requiring ICU Care

From 2009 influenza hospital discharge data, we estimated that 18% of patients required ICU care during the peak week. Texas DSHS reported that 23% of the 2,030 confirmed influenza A(H1N1)pdm09 patients requiring hospitalization in Texas during October–December 2009 required ICU care (*19*). For moderate and severe planning scenarios, the US Homeland Security Council (HSC) (*21*) uses an ICU proportion of 15% for the overall pandemic and 25.7% for the peak week. For seasonal influenza, CDC’s FluSurge 2.0 (*22*,*23*) assumes that a baseline of 15% of admitted influenza patients require ICU care; HHS makes similar assumptions (*2*) (Technical Appendix Table 3). On the basis of these data and reports, we assumed peak-week ICU proportions of 20% during a mild pandemic and 25% during moderate and severe pandemics.

#### Proportion of ICU Patients Requiring Ventilation

FluSurge 2.0 assumes 50% of patients with seasonal influenza admitted to the ICU require ventilation (*22*). HSC assumes 50% throughout a pandemic (*21*), and HHS uses 50.4% for a moderate scenario and 50% for a severe scenario (*2*) (Technical Appendix Table 3). We assumed that 50% of patients in the ICU who have pandemic influenza require ventilation across all scenarios.

#### Proportion of Ventilated Patients Requiring 2 Weeks of Ventilation

FluSurge 2.0 (*22*) assumes that ventilatory support of ILI patients lasts 10 days. We have weekly time resolution and assumed 60% of patients receiving ventilatory support require only 1 week, and the remaining 40% require a second week.

### Simulating Pandemic Scenarios

We generated a mild scenario by fitting our forecasting model to hospital discharge data for the 2009 pandemic. Because comparable data are not available from 1957 and 1968 (moderate) and 1918 (severe), we scaled the 2009 estimates to model these scenarios. HHS (*2*) and HSC (*21*) use similar pandemic scaling factors, except HSC rates for hospitalization, ICU care, and mechanical ventilation are ≈17% and 14% lower than HHS rates for moderate and severe scenarios, respectively. (See [*24*] for scaling methods for an emerging pandemic.) CDC’s median estimate of hospitalizations for influenza A(H1N1)pdm09 (April 2009–April 2010) is 275,000. Combining this with the HHS scenario (Technical Appendix Table 3), we scaled our mild pandemic hospitalization estimates by 865,000/275,000 = 3.14 and 9,900,000/275,000 = 36 to model moderate and severe scenarios, while preserving the variability, spatial correlation, and temporal correlation estimated for 2009.

## Results

**Figure 2:**
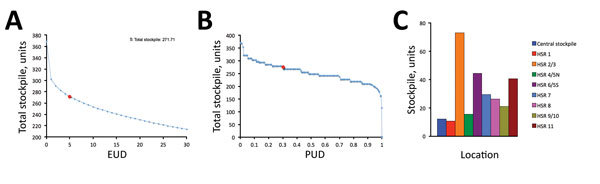
Optimal ventilator stockpiles for a mild pandemic scenario, Texas, USA. The total size of the optimal stockpile, summed across the central and 8 HSR stockpiles, decreases as risk tolerance increases. Risk for unmet demand for ventilators is quantified as the expected number of hospitalized influenza patients statewide not receiving necessary ventilation (EUD) (A) and the probability of at least 1 hospitalized patient in Texas not receiving necessary ventilation (PUD) (B). We optimized directly for EUD and calculated PUD post hoc. Red circles indicate EUD/PUD of 5 patients. C) Optimal allocation among central and regional sites when EUD is set to 5 patients, equivalent to a stockpile of 272 ventilators. EUD, expected unmet demand; PUD, probability of unmet demand; HSR, health service region.

Under the mild pandemic scenario, recommended stockpiles ranged from 200 to 400 ventilators (Figure 2, panel A). For example, if we specify the risk tolerance to be an EUD of at most 5 patients, then the recommended stockpile is 272 ventilators, including a central stockpile of 12. The PUD for this scenario, which is computed post hoc, is 30% (Figure 2, panel B). Thus, if the public health department builds the recommended central and local stockpiles, it can expect that no more than 5 patients statewide will go without ventilation, and a 70% chance exists that no demand anywhere will be unmet. As the risk tolerance decreases from an EUD of 5, the recommended stockpile grows sharply; as the EUD increases, the stockpile decreases nearly linearly (Figure 2, panel A). Ventilators are allocated primarily to local sites rather than to the central stockpile (Figure 2, panel C). 

**Figure 3:**
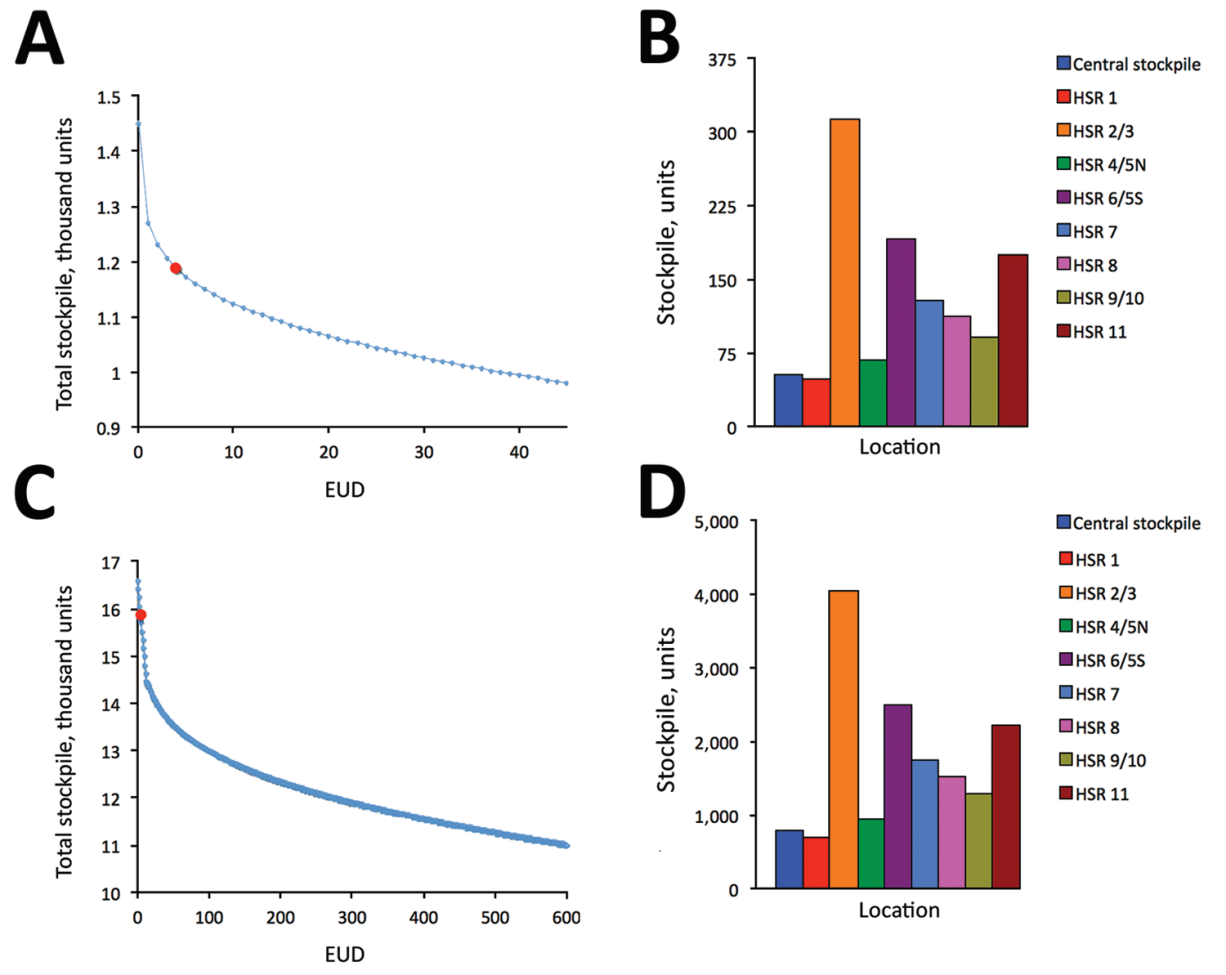
Optimal ventilator stockpiles for moderate and severe pandemic scenarios, Texas, USA. The total size of the required stockpile, summed across the central and 8 HSR stockpiles, decreases as risk tolerance (EUD) increases, for both moderate (A) and severe (C) pandemic scenarios. For an EUD of 5 patients (red circles), total stockpiles would be 1,172 (A) and 15,697 (C); optimal allocations to central and regional stockpiles are shown for moderate (B) and severe (D) scenarios. EUD, expected unmet demand; PUD, probability of unmet demand; HSR, health service region.

The optimal stockpile allocations under moderate and severe pandemic scenarios are qualitatively, but not quantitatively, similar (Figure 3). With an EUD tolerance of 5 patients, the recommended stockpiles increase to 1,172 and 15,697 ventilators for moderate and severe scenarios, respectively. These stockpiles scale roughly according to our assumptions that moderate and severe pandemics have hospitalization rates of 3.14 and 36 times higher than the mild pandemic, respectively, and that the fraction of hospitalized patients requiring ICU admission increases from 20% in the mild scenario to 25% in the other scenarios. Specifically, peak ventilator demand increases by factors of (0.25/0.20) × 3.14 = 3.93 and (0.25/0.20) × 36 = 45 from the mild to moderate and severe scenarios, respectively. This scaling would exactly predict how stockpiles would grow if we increased the risk tolerance by factors of 3.93 and 45. However, we fixed the EUD limit to 5 patients, so stockpile growth exceeds these scaling factors.

### Sensitivity Analysis

We assessed the sensitivity of the recommended stockpiling strategies to several factors. For a fixed risk tolerance (EUD), increasing the proportions of hospitalized patients requiring ICU admission and ventilation results in comparable increases in the recommended stockpiles. However, increasing the proportion of patients requiring 2 weeks of ventilation (rather than just 1) produces a slightly more complicated effect. Because the demand at peak week will depend on both established and newly admitted patients, increasing the 2-week proportion from 0 to 1 might not exactly double the demand. Based on 2009 pandemic hospitalization data, peak-week mean demand across Texas is expected to increase by a factor of 1.42 when the 2-week proportion increases from 0.4 to 1. The recommended stockpile grows accordingly. Under the mild pandemic scenario, the stockpile grows by a factor of 1.38 for an EUD near 0 ventilators and 1.42 for an EUD close to 5 ventilators.

We also varied the wastage rate for centrally held ventilators and the region-to-region correlation in peak demand. The baseline wastage of 0.2 means that 1 in 5 ventilators distributed from the central stockpile is not used effectively. This wastage contributes to relatively small recommended central stockpiles (e.g., just 4.4% of the total stockpile under the mild scenario with an EUD of 5 ventilators). As the wastage rate decreases, the central allocation slowly increases (Table 2; Technical Appendix Figure 2). The benefit of risk pooling through a central stockpile also grows as the region-to-region correlation in peak demand shrinks (Table 2; Technical Appendix Figure 3).

**Table 2:** Central stockpile size, as a percentage of total stockpile, as a function of wastage and region-to-region correlation in peak ventilator demand during an influenza pandemic, Texas, USA*

Wastage, %	Central stockpile, %		
Region-to-region correlation = 0.55	Region-to-region correlation = 0.70	Region-to-region correlation = 0.85
40	2.8	1.5	0.2
30	4.7	2.9	0.9
20	7.0	**4.4**	2.0
10	9.8	6.7	3.5
1	18.0	13.5	10.1
0.5	25.4	20.6	17.0
0.3	32.8	27.4	22.7
0.2	48.2	43.9	39.3
0.1	100	100	100

*The results in this table are based on a mild pandemic scenario and a limit on expected unmet demand of 5 patients. Bold indicates the baseline value.

### Retrospective Analysis of 2009 Pandemic

During the 2009 pandemic, hospitals across Texas held an estimated 3,730 ventilators. When aggregated by region, the 8 HSRs had stockpiles ranging from 151 to 1,233 ventilators (Technical Appendix Table 1). Under mild and moderate pandemic scenarios, we projected expected statewide demands for 230 and 903 ventilators, respectively, with each HSR holding a stockpile at least 6 SD above the forecasted mean demand. Given this ample regional surge capacity, there would have been no need for central stockpiling. Under the severe scenario, however, the projected statewide demand is 10,333 ventilators, far exceeding 2009 stockpiles.

## Discussion

Central stockpiles can save costs but are advisable only when spatial correlation in peak demand is sufficiently low and stockpile deployment is sufficiently reliable. Data from Texas suggest that influenza peaks strongly correlate across regions. Such synchrony undercuts the risk-pooling benefits of central stockpiles. Furthermore, successful deployment requires not only central maintenance and physical transportation of ventilators to patients in need, but also healthcare facilities and clinicians trained to administer and troubleshoot available ventilator models, which might differ from those held locally. Pandemic-related staff absenteeism might exacerbate this challenge. Our model incorporates this limitation by assuming that fraction of stockpiled ventilators are wasted. When we considered a plausible wastage parameter of 20% (based on discussions with Texas DSHS about likely impediments to successful deployment), the model recommended that <10% of ventilators be held centrally.

The recommended allocations among central and local stockpiles hinge critically on the relative efficiencies of a local versus central stockpile, which are largely unknown and perhaps changing to favor central stockpiles as delivery technology continues to improve. We made the simplifying assumption that locally held ventilators are perfectly matched to patients, and we considered a range of potential wastage rates for centrally held ventilators. In general, the more reliable central stockpile deployment, the more advisable a central stockpile. For example, assuming only 0.1% wastage, we found that that all ventilators should be held centrally, regardless of spatiotemporal correlations in peak demand (Table 2). Thus, as deployment and local capacities continue to improve, distance will become less of an issue, and the advantages of central stockpiles might outweigh their shortcomings.

Our surprisingly small central allocation stems from 2 additional factors. First, the uncertainty in our estimates of peak hospitalizations, based on 2009 pandemic data, is relatively low. Across Texas’ 8 HSRs, the coefficient of variation (measuring the level of uncertainty) in peak demand for ventilators ranged from 0.17 to 0.36 and averaged 0.24 (online Technical Appendix Table 4). When we increase these coefficients governing uncertainty 3-fold, the recommended central stockpile increases only from 4.4% to 10% of the total, assuming a mild pandemic and a risk tolerance (EUD) of 5 untreated patients. Second, the small central allocation depends on the risk tolerance. As the risk tolerance shrinks from an EUD of 5 patients, both the number of ventilators in the total stockpile and the percentage held centrally grow (Technical Appendix Figure 3). Still, even at tighter risk tolerances and a smaller region-to-region correlation in peak demand of 0.55, the central stockpile is <10%.

Our retrospective analysis of the 2009 influenza A(H1N1) pandemic in Texas suggests that hospitals had enough ventilators on hand to treat all patients requiring mechanical ventilation throughout the pandemic. Although these quantities are expected to suffice for a moderate (1957- and 1968-like) pandemic, in which hospitalization rates roughly triple, they would fall far short in a severe (1918-like) pandemic. If we optimistically assume perfect deployment, that is, 0 wastage, by assuming timely delivery, adequately trained and available staff (respiratory therapists, nurses, and physicians), sufficient space to care for a potentially large number of patients, and requisite ancillary equipment and supplies, then even a central stockpile of 8,900 ventilators in Texas—the total number of SNS ventilators in 2010 (*9*)—would fall short, with an expected unmet demand of 576 patients.

Technical Appendix Forecasting peak-week demand for ventilators, optimization model for stockpiling, and additional tables and figures.
