# Supplementary material for: Stockpiling Ventilators for Influenza Pandemics
Source: Emerg Infect Dis. 2017 Jun;23(6):914–21. doi: 10.3201/eid2306.161417 (PMC5443432; doi:10.3201/eid2306.161417)
Supplement: Technical Appendix — Forecasting peak-week demand for ventilators, optimization model for stockpiling, and additional tables and figures. [file 16-1417-Techapp-s1.pdf]

Article DOI: http://dx.doi.org/10.3201/eid2306.161417

Stockpiling Ventilators for Influenza Pandemics

Technical Appendix

Materials and Methods 1: Forecasting Peak-Week Demand for Ventilators

We describe here the mathematical and technical details of the model used to forecast influenza-like-illness (ILI) hospitalizations. The purpose of the dynamic linear model (DLM) that we formulate is to generate accurate estimates of ILI hospitalizations as, and when, new information on the predictor variables becomes available. Formally embedding this "learning from experience" notion into the mathematical framework is one of the key merits of the Bayesian updating of the stochastic parameters in a DLM.

Forecasting of Hospitalizations

The predictors we use for forecasting include ILINet weekly reports for the state of Texas, and 4 time-indicator variables to account for the seasonality effect on ILI hospitalizations. We group months as September–October (S–O), November–December (N–D), January–February (J–F), and March–April (M–A). So, the corresponding indicator variable takes value 1 or all indicator variables are 0 for May–August. We considered models that also included predictors of school calendars, a humidity index, and Google Flu Trends, but for the significant look-ahead period we require for stockpiling ventilators these variables did not add significant predictive power to the model. Before proceeding with the details of the forecasting model for ILI hospitalizations, we specify notation.

Notation

h_t: dependent variable of ILI hospitalizations at time t (weeks)

z_t: independent variable of ILINet weekly reports at time t

γ_t^i: time indicators for season i at time t, $i \in I = \{1,2,3,4\}$

$$\gamma_t^1 = \begin{cases} 1 & \text{if } t \text{ is in S-O} \\ 0 & \text{otherwise} \end{cases} \qquad \gamma_t^2 = \begin{cases} 1 & \text{if } t \text{ is in N-D} \\ 0 & \text{otherwise} \end{cases}$$

$$\gamma_t^3 = \begin{cases} 1 & \text{if } t \text{ is in J-F} \\ 0 & \text{otherwise} \end{cases} \qquad \gamma_t^4 = \begin{cases} 1 & \text{if } t \text{ is in M-A} \\ 0 & \text{otherwise} \end{cases}$$

We could formulate a static multiple linear regression model to study ILI hospitalizations in the following manner:

$$h = \beta^0 + \beta z + \sum_{i \in I} \alpha^i \gamma^i . \tag{1}$$

However, to incorporate the evolution of the predictors over time, which has significant importance in forecasting of ILI hospitalizations, we instead posit a dynamic linear regression model:

$$h_t = \beta_t^0 + \beta_t z_t + \sum_{i \in I} \alpha_t^i \gamma_t^i . \tag{2}$$

The critical difference between equations (1) and (2) is that the regression parameters are no longer static, evidenced by introducing the time subscript t in equation (2). The estimation of the random parameters in equation (2) can be performed recursively using the Kalman filter (*1*). Let $(H_t)_{t\geq 1}$ be the time series of ILI hospitalizations influenced by the nonrandom regression parameters corresponding to the independent variables; i.e., the regression coefficients for ILINet reports and the 4 time-indicators. The independent variables form the regression vector F_t at time t while their coefficients are represented by the state vector θ_t. The state matrix G_t is the evolution of the state vector through time. By introducing Gaussian measurement errors, v_t, and Gaussian state evolution errors, w_t, the dynamic linear model is given by:

$$H_t = F_t^T \theta_t + v_t, \qquad v_t \sim N(0, V_t)$$

$$\theta_t = G_t \theta_{t-1} + w_t, \qquad w_t \sim N(0, W_t).$$

Here H_t, v_t, and V_t are univariate, while F_t, θ_t, and w_t are p-dimensional vectors where, in our setting, p = 6, including the intercept term. The matrices G_t and W_t are $p \times p$ in dimension.

State Estimation and Observation Forecasting

The recursive procedure for updating the state vector θ_t and forecasting the response variable H_t of the dynamic linear model now follows. At time $t-1$, for some mean m_{t-1} and covariance matrix C_{t-1} the information about the state θ_{t-1} is presented with the posterior distribution:

$$(\theta_{t-1} | h_{1:t-1}) \sim N(m_{t-1}, C_{t-1}).$$

The recursive procedure starts at time 0 by choosing m_0 and C_0 to be the best guess regarding the mean and variance of the state vector. We use a subset of the data in the simple regression model (1) to construct the prior information about m_0 and C_0. Through direct application of Bayes' theorem, we obtain that the prior distribution of θ_t given $h_{1:t-1}$ is Gaussian, i.e., $(\theta_t | h_{1:t-1}) \sim N(a_t, R_t)$, with a_t and R_t being:

$$a_t = \mathrm{E}(\theta_t | h_{1:t-1}) = G_t m_{t-1}$$

$$R_t = Var(\theta_t | h_{1:t-1}) = G_t C_{t-1} G_t^T + W_t.$$

Next, the 1-step-ahead predictive distribution of H_t given $h_{1:t-1}$ is also Gaussian, i.e., $(H_t | h_{1:t-1}) \sim N(f_t, Q_t)$, with f_t and Q_t as follows:

$$f_t = \mathrm{E}(H_t | h_{1:t-1}) = F_t^T a_t$$

$$Q_t = Var(H_t | h_{1:t-1}) = F_t^T R_t F_t + V_t.$$

After obtaining the observation h_t, the filtering distribution of θ_t is, again, Gaussian, i.e., $(\theta_t | h_{1:t}) \sim N(m_t, C_t)$. The parameters m_t and C_t can be computed as follows:

$$m_t = \mathrm{E}(\theta_t | h_{1:t}) = a_t + R_t F_t e_t / Q_t$$

$$C_t = Var(\theta_t | h_{1:t}) = R_t - R_t F_t F_t^T R_t / Q_t,$$

where $e_t = H_t - f_t$ is the forecast error. Our discussion here follows the book (*1*), which we refer to for further details.

Multiple Steps Ahead Forecasting

For the purpose of producing demand scenarios for our optimization model, we must forecast hospitalizations and in turn, ventilator demand, many weeks into the future. Suppose we wish to forecast k weeks ahead. With the observed values of $h_{1:t}$, we can also forecast the future values of the state vector θ_{t+k} and the observation H_{t+k}. Let $a_t(0)=m_t$ and $R_t(0)=C_t$. Then, for $k\geq 1$, the distribution of θ_{t+k} given $h_{1:t}$ is Gaussian, i.e., $(\theta_{t+k}|h_{1:t})\sim N(a_t(k),R_t(k))$, with $a_t(k)$ and $R_t(k)$ being:

$$a_t(k)=\mathrm{E}(\theta_{t+k}|h_{1:t})=G_{t+k}a_t(k-1)$$

$$R_t(k)=Var(\theta_{t+k}|h_{1:t})=G_{t+k}R_t(k-1)G_{t+k}^T+W_{t+k}.$$

The distribution of H_{t+k} given $h_{1:t}$ is also Gaussian, i.e., $(H_{t+k}|h_{1:t})\sim N(f_t(k),Q_t(k))$, with $f_t(k)$ and $Q_t(k)$ as follows:

$$f_t(k)=\mathrm{E}(H_{t+k}|h_{1:t})=F_{t+k}^T a_t(k)$$

$$Q_t(k)=Var(H_{t+k}|h_{1:t})=F_{t+k}^T R_t(k)F_{t+k}+V_{t+k}.$$

We use 1 year of historical seasonal influenza data to construct the prior for m_0 and C_0, and we use 2009 pandemic data to fit the model and forecast k = 40 weeks into the future.

From Hospitalizations to Peak-Week Demand for Ventilators

We index the health service regions (HSRs) in Texas by $r\in R$. The DLM predicts hospitalizations on a weekly basis for each of the 8 HSRs in the form of a multivariate Gaussian distribution, providing the means ($f_{r,t}$) and variances ($Q_{r,t}$) for each region. We estimate the region-to-region correlations (ρ_{HSR}) using historical data, and we assume this correlation to be identical for each pair of regions. To estimate the peak-week demand for ventilators from the forecasted hospitalizations, we employ 4 additional parameters: 1) p_i, the proportion of hospitalized ILI patients requiring ICU care; 2) p_v, the proportion of ICU patients requiring ventilation; 3) p_{tw}, the proportion of ventilated patients requiring 2 weeks of ventilation, under

the assumption that at most 2 weeks is needed; and, 4) $\rho_{r,t}$, 1-week lagged temporal correlation in ILI hospital admission in region r at time t generated by the DLM.

We calculate the mean weekly demand for ventilators in region r at time t as follows:

$$\mu_{r,t} = (p_{tw} f_{r,t-1} + f_{r,t}) p_i p_v. \quad (3)$$

We obtain the corresponding variance of weekly demand for ventilators, involving temporal correlation ($\rho_{r,t}$), as follows:

$$\sigma^2_{r,t} = Q_{r,t} p_i^2 p_v^2 + p_{tw}^2 Q_{r,t-1} p_i^2 p_v^2 + 2\rho_{r,t} p_{tw} \sqrt{Q_{r,t-1} p_i^2 p_v^2 Q_{r,t} p_i^2 p_v^2}.$$

We choose the peak-week demand in a region as the week with the largest mean according to equation (3). With the estimated region-to-region correlation (ρ_{HSR}), we employ a standard Monte Carlo sampling algorithm (*2*) to generate independent and identically distributed (i.i.d.) samples of peak-week demand for ventilators as input to the optimization model, which we describe next.

Materials and Methods 2: Optimization Model for Stockpiling

Two-Stage Optimization Model

To optimize stockpiling decisions, we construct a 2-stage stochastic program. We index the regional sites by $r \in R$. The value of the central stockpile, x, and the value of the stockpiles at each site, $s = (s_r)_{r \in R}$, must be selected before observing the demand for ventilators $d(\omega) = (d_r(\omega))_{r \in R}$. The decision to ship ventilators from the central stockpile to site r is captured by decision variable $y_r(\omega)$, and this decision is made after observing the demand realization, indexed by $\omega \in \Omega$. In addition, if $y_r(\omega)$ ventilators are shipped, then $wy_r(\omega)$ represents the number of ventilators wasted so that only $(1-w)y_r(\omega)$ ventilators can be used at site r. Hence, the model seeks a balance between 1) the flexibility permitted by holding ventilators centrally so that they can be distributed to where they are needed most, and 2) the fact that locally held ventilators are more effective than those shipped from the central stockpile after a pandemic begins. The optimization model for stockpiling is as follows:

$$\min_{x,s,y} x+\sum_{r\in R} s_r \tag{4a}$$

s.t. $$\sum_{r\in R} y_r(\omega)\le x, \forall \omega \in \Omega \tag{4b}$$

$$\mathrm{E}_\omega\left[\sum_{r\in R}\left[\left[d_r(\omega)-s_r\right]^+ -(1-w)y_r(\omega)\right]^+\right]\le L \tag{4c}$$

$$x\ge 0, s_r \ge 0, y_r(\omega)\ge 0, \forall r \in R, \omega \in \Omega. \tag{4d}$$

The objective function we minimize in (4a) is the total stockpile of central and regional ventilators. Constraint (4b) says that the total number of ventilators distributed from the central stockpile to the sites cannot exceed the number of ventilators stockpiled centrally. We let $\left[d_r(\omega)-s_r\right]^+ = \max\left\{d_r(\omega)-s_r, 0\right\}$ represent the amount by which peak demand for ventilators exceeds the existing supply at site r under scenario ω, and $\sum_{r\in R}\left[\left[d_r(\omega)-s_r\right]^+ -(1-w)y_r(\omega)\right]^+$ represents the total shortfall of ventilators statewide after distributing the central stockpile under scenario ω. Thus constraint (4c) ensures that the expected shortfall of ventilators over all sites does not exceed the limit, L. Constraint (4d) enforces non-negativity for each decision variable. Note that $d(\omega)$, w, and L are input data, and $y(\omega)=(y_r(\omega))_{r\in R}$ are decision variables. By prespecifying the values of x, s, or neither, 3 variations of the model can be formulated with respect to stockpiling decisions:

1. Given existing stockpiles at the regional sites, optimize the number of centrally held ventilators.
2. Given an existing central stockpile, optimize the number of ventilators at each site.
3. Jointly optimize the central and regional stockpiles, allowing us to assess the advantages of stockpiling ventilators centrally versus at the sites.

Model (4) is stated in the form of the third variation above, but the first 2 variations can also be handled by fixing decision variables s or x, respectively, to prespecified values.

We cannot solve model (4) directly for the following reasons. The summed shortfall of ventilators, i.e., $\sum_{r\in R}\left[\left[d_r(\omega)-s_r\right]^+ -(1-w)y_r(\omega)\right]^+$, is a nonstandard random variable due to

the 2 positive-part operators within the summation, even though $d(\omega)$ has the form of a multivariate normal distribution. More importantly, the decision variables, $y(\omega)$, representing shipments to the sites, adapt to the demand realization under scenario ω, increasing the model's complexity. Hence, below we create a sampling-based variant of model (4), using a standard Monte Carlo sampling algorithm (*2*) to generate a set of i.i.d. samples of peak demands from the multivariate normal distribution we describe earlier.

A Monte Carlo Approximation to the Optimization Model

Let $i=1,\ldots,n$ index the sampled scenarios. Our sampling-based variant of model (4) is as follows:

$$\min_{x,s,y,u,v} x+\sum_{r\in R} s_r \tag{5a}$$

s.t.

$$\sum_{r\in R} y_r^i \le x, \forall i=1,2,\ldots,n \tag{5b}$$

$$u_r^i \ge d_r^i - s_r, \forall r\in R, i=1,2,\ldots,n \tag{5c}$$

$$v_r^i \ge u_r^i - (1-w)y_r^i, \forall r\in R, i=1,2,\ldots,n \tag{5d}$$

$$\frac{1}{n}\sum_{i=1}^{n}\sum_{r\in R} v_r^i \le L \tag{5e}$$

$$x\ge 0, s_r \ge 0, y_r^i \ge 0, u_r^i \ge 0, v_r^i \ge 0, \forall r\in R, i=1,2,\ldots,n. \tag{5f}$$

The objective function in (5a) is identical to that in (4a). Constraint (5b) is analogous to constraint (4b), where we add index i to variable y_r because shipments from the central stockpile to the sites occur after observing the demand realization. In constraint (5c), $d^i=(d_r^i)_{r\in R}, i=1,2,\ldots,n$, are the samples of ventilator demands, and in constraint (5d), $(1-w)$ is the proportion of centrally held ventilators dispatched to the site that can be used. These 2 constraints take care of the 2 positive-part operators in constraint (4c) by using 2 new decision variables, u_r^i and v_r^i. Given that these variables capture the positive parts, constraint (5e) is analogous to constraint (4c), and constraint (5f) again captures non-negativity of all decision variables. While we state models (4) and (5) for a fixed value of L, we view this as a bi-criteria model in which we can explore the tradeoff between the cost of the total stockpile (which we assume is proportional to the number of ventilators) and the limit on expected shortfall (L).

References

1. Petris G, Petrone S, Campagnoli P. Dynamic linear models with R. New York: Springer; 2009.

2. Devroye L. Non-uniform random variate generation. New York: Springer-Verlag; 1986.

3. US Department of Health and Human Services (HHS). HHS pandemic influenza plan. 2005 [cited 2016 Jun 16]. http://www.flu.gov/planning-preparedness/federal/hhspandemicinfluenzaplan.pdf

4. Texas Department of State Health Services. The health service regions. 2014 [cited 2016 Jun 16]. http://www.dshs.state.tx.us/regions/state.shtm

Technical Appendix Table 1. Existing regional stockpiles of ventilators in the state of Texas*

Region	No. of existing ventilators
HSR 1	151
HSR 2/3	1,233
HSR 4/5N	247
HSR 6/5S	742
HSR 7	247
HSR 8	458
HSR 9/10	287
HSR 11	365

*HSR, health service region.

Technical Appendix Table 2. Temporal correlation in the dynamic linear model between consecutive weeks, April–December 2009*

Region	Minimum	Peak week	Median	Maximum
HSR 1	0.38	0.38	0.44	0.46
HSR 2/3	0.08	0.11	0.28	0.28
HSR 4/5N	0.19	0.19	0.23	0.24
HSR 6/5S	0.32	0.34	0.64	0.65
HSR 7	0.19	0.20	0.33	0.35
HSR 8	0.16	0.16	0.29	0.30
HSR 9/10	0.08	0.12	0.42	0.43
HSR 11	0.07	0.07	0.20	0.21

*When the peak-week correlation is not the minimum correlation over the 9 months, the minimum instead occurs the week before the peak week. HSR, health service region.

Technical Appendix Table 3. Number of illnesses, healthcare utilization, and deaths associated with moderate and severe pandemic influenza scenarios*

Characteristics	Moderate (1957- and 1968-like), no. (%)	Severe (1918-like), no. (%)
Illness	90 million (30)	90 million (30)
Outpatient medical care	45 million (50)	45 million (50)
Hospitalization	865,000	9,900,000
ICU care	128,750	1,485,000
Mechanical ventilation	64,875	742,500
Deaths	209,000	1,903,000

*Source: (*3*). ICU, intensive care unit.

Technical Appendix Table 4. Estimated regional peak-week demand for ventilators in the mild scenario*

Region	Mean, units	± SD, units	Coefficient of variation
HSR 1	8.59	3.09	0.36
HSR 2/3	66.83	11.31	0.17
HSR 4/5N	12.93	3.48	0.27
HSR 6/5S	40.2	7.79	0.19
HSR 7	25.14	6.01	0.24
HSR 8	22.41	5.29	0.24
HSR 9/10	17.55	4.66	0.27
HSR 11	35.97	7.70	0.21

*These estimates are based on April–December 2009 hospital discharge data in Texas. All the regional peak demands have a coefficient of variation <0.40, although the means range from 8.59 to 66.83. HSR, health service region.

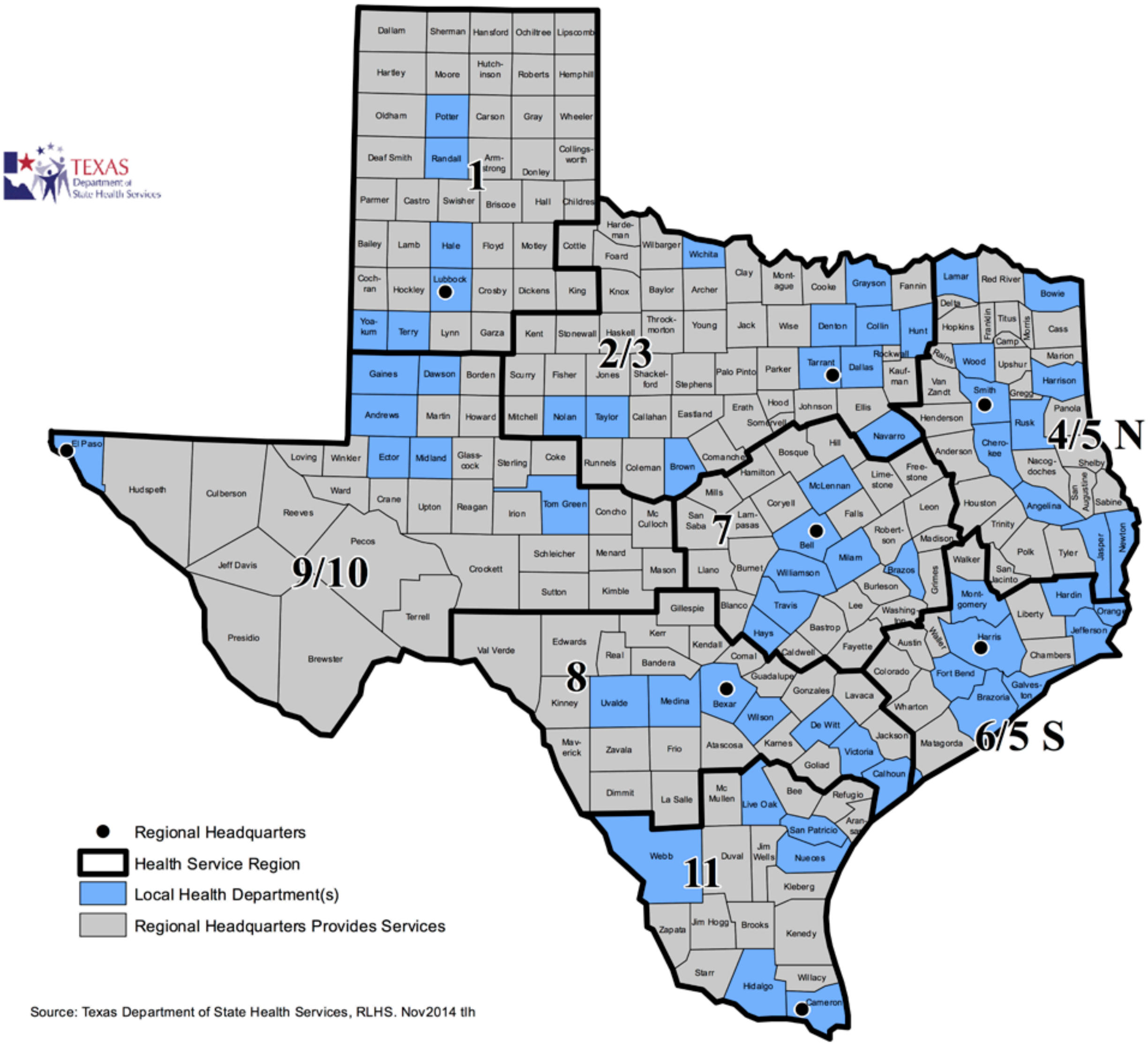

Technical Appendix Figure 1. The 8 health service regions in Texas. Source: (*4*).

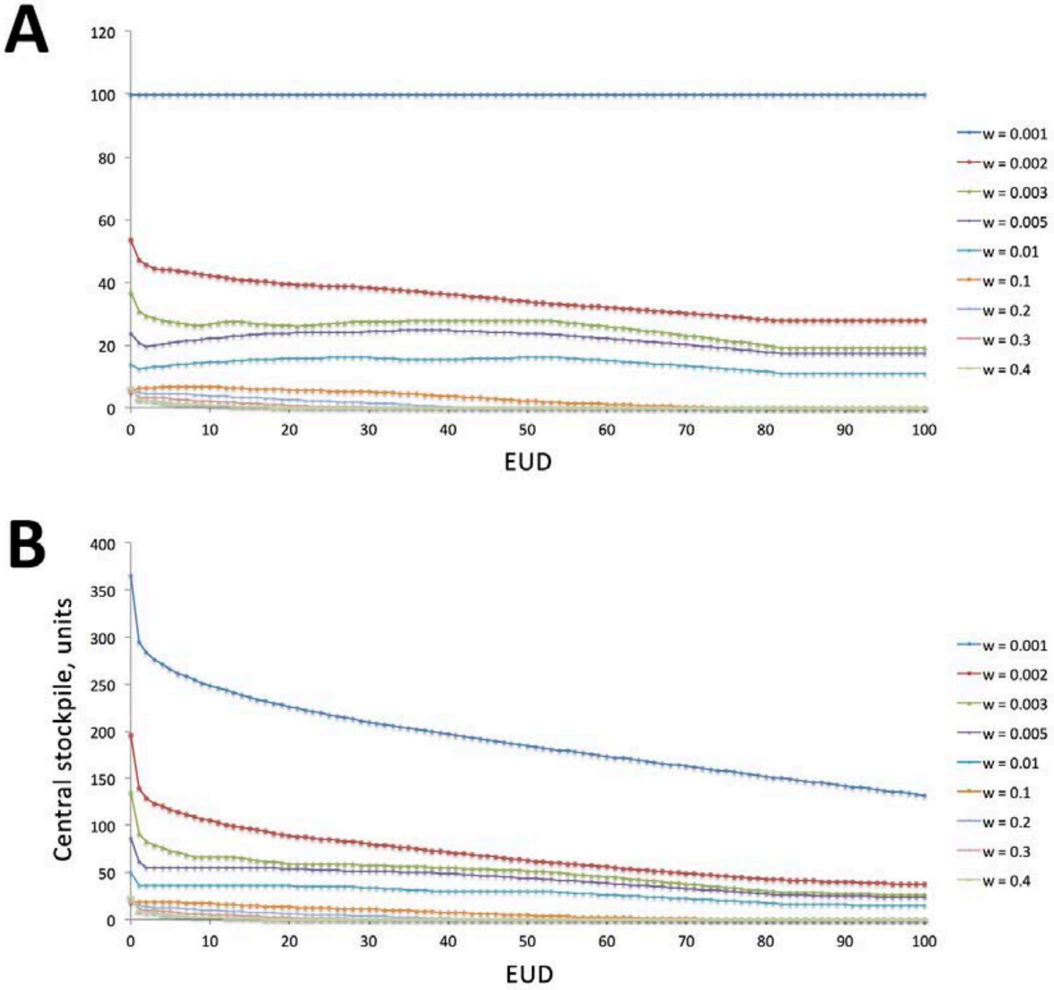

Technical Appendix Figure 2. The central stockpile versus EUD for various values of the wastage parameter (w) for the mild influenza pandemic scenario, Texas, USA. The baseline result corresponds to w = 0.2, or 20%. A) Change in the percentage of the stockpile held centrally with the growth of EUD. B) Change in the number of ventilators held in the central stockpile. Mean peak-week demand, summed across all regions, is ≈230 ventilators in the mild scenario. EUD, expected unmet demand.

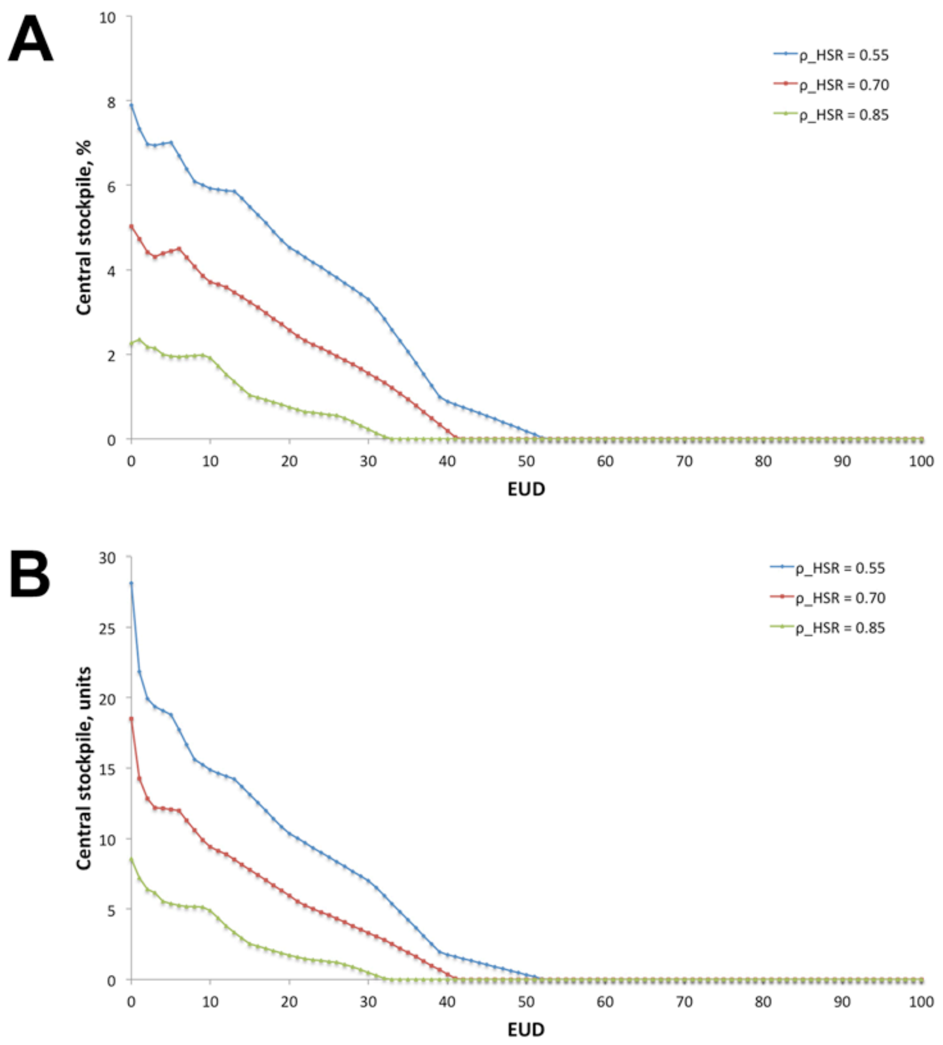

Technical Appendix Figure 3. The central stockpile versus EUD for various values of the region-to-region correlation coefficient (ρ_{HSR}) under the mild influenza pandemic scenario, Texas, USA. The baseline result corresponds to ρ_{HSR} = 0.70. A) Change in the percentage of the stockpile held centrally with the growth of EUD. B) Change in the number of ventilators held in the central stockpile. EUD, expected unmet demand.
